# Colon Cancer Rates Among Asian Americans: A 2017–2021 Epidemiological Analysis

**DOI:** 10.3390/cancers16244254

**Published:** 2024-12-20

**Authors:** Candice Do, Wei-Chen Lee, Christopher Huy D. Doan, Cathy Z. Xie, Kendall M. Campbell

**Affiliations:** 1 John Sealy School of Medicine, University of Texas Medical Branch, Galveston, TX 77555, USA; cado@utmb.edu (C.D.); cddoan@utmb.edu (C.H.D.D.); 2 Department of Family Medicine, University of Texas Medical Branch, Galveston, TX 77555, USA; czxie@utmb.edu (C.Z.X.); kemcampb@utmb.edu (K.M.C.)

**Keywords:** Asian American, colon cancer, social determinants of health, disparities

## Abstract

**Background:** Colon cancer (CC) is a significant public health concern. With Asian Americans (AA) representing a rapidly growing demographic in the United States, our study examined CC prevalence among AA. **Methods:** The study merged 2017–2021 Medical Expenditure Panel Survey and County Health Ranking. Our analysis calculated age-adjusted CC rates and examined prevalence across states. Regression analyses were conducted to study county-level risk factors of CC. **Results:** The CC age-adjusted rate among AA increased five-fold, from 155 per 100,000 in 2017 to 753 per 100,000 in 2021. State-level disparities revealed the highest CC prevalence in Arkansas, Rhode Island, and New Hampshire. Not speaking other languages and having insurance were significantly associated with higher CC rates, suggesting barriers to preventions and higher use of screening (*p* < 0.05). County-level analysis identified higher CC prevalence in regions with greater socioeconomic advantage (*p* < 0.05). Socioeconomic advantage seemed to facilitate higher screening rates, translated into higher CC rates. **Conclusions:** Our findings underscore the need for early preventions to address rising CC rates among AA. Future research should also explore geographic to better understand the disparities in CC risk.

## Simple Summary

Colon cancer is an increasing health concern, especially for Asian Americans. Our study analyzed recent data to understand colon cancer prevalence and risk factors in Asian Americans across different states and counties. We found that colon cancer prevalence among Asian Americans increased significantly between 2017 and 2021, and the highest rates were in Arkansas, Rhode Island, and New Hampshire. Speaking only English and having health insurance were associated with higher prevalence of colon cancer, possibly due to greater access to screening. Communities with greater socioeconomic resources had higher rates of colon cancer, likely due to better access to early screenings. These findings highlight the importance of preventive efforts to address the increased colon cancer rates in the Asian American population. Future studies should examine geographic and community-level factors contributing to these disparities.

## 1. Introduction

Colon cancer (CC) is the third most common cancer in the United States, but it can be effectively treated if detected early through regular screening [1]. However, an estimated 106,590 new cases of CC are expected in the United States in 2024, highlighting the ongoing need for screening and prevention efforts [2]. Despite the increasing representation of racial and ethnic minorities in medical research, a disparity remains in the availability of studies specifically addressing CC prevalence in Asian American populations.

Asian Americans (AAs), comprising about 6.0% of the U.S. population (nearly 20 million individuals), represent a diverse group, with at least 21 subgroups [3]. While the overall colorectal cancer incidence in AAs (28.6 per 100,000 people from 2015 to 2019) is lower than that of other racial groups, disparities remain. For example, Japanese Americans have higher colorectal cancer incidence rates than White Americans (25% higher in men and 11% higher in women) [4,5,6]. Although CC is frequently grouped with rectal cancer under the term colorectal cancer, they differ notably in terms of risk factors, anatomic locations, and surgical treatments [7]. Focusing specifically on CC allows us to address unique disparities in screening and prevention, particularly in the AA population, where differences in CC prevalence may be overlooked when combined with rectal cancer.

Risk factors that may increase CC risk among Asian immigrants and subsequent generations include acculturation, Western diets, and sedentary lifestyles [8]. Immigration status has been associated with colorectal cancer, as Asian immigrants in the U.S. have colorectal cancer rates higher than those of their country of origin, likely due to changes in diet (including increased red meat consumption), having a sedentary lifestyle, and obesity [8]. Genetics also play a role in susceptibility, with South Asians potentially having a lower incidence due to protective genetic factors [5]. Phenotypic differences in CC formation have been noted, with AAs being more likely than White Americans to develop left-sided adenomas and less likely to have sessile serrated polyps [9]. However, most studies do not disaggregate CC from rectal cancer, limiting our understanding of CC-specific risks within AA subgroups.

Barriers to healthcare access further exacerbate disparities in CC screening rates among AAs. The American Cancer Society reports that only 50% of AAs aged 45 and older undergo colorectal cancer screening, compared to 61% of White and Black Americans and 52% of Hispanics [6]. AA people who have lived in the United States for more than 15–20 years and have higher levels of acculturation tend to have increased colorectal cancer screening rates [9], while language barriers, unfamiliarity with the U.S. healthcare system, and lower health literacy hinder screening efforts among recent immigrants [10,11].

Geographical disparities in colorectal cancer incidence exist within the AA population. From 2006 to 2016, colorectal cancer incidence in AAs declined in the West but remained unchanged in the Midwest and South [12]. This variation may be attributed to lower socioeconomic status, lack of insurance, and risk factors such as diet and physical inactivity. While these studies focus broadly on colorectal cancer, the distinct regional and socioeconomic challenges likely apply to CC as well.

Our study utilizes data from the Medical Expenditure Panel Survey (MEPS), which provides comprehensive information on health utilization, costs, insurance, and quality of healthcare. By analyzing annual trends in CC prevalence among AAs across different age groups and geographic regions and integrated state- and county-level socioeconomic data, our study aims to provide insights into factors influencing CC prevalence in this demographic. We excluded rectal cancer from our analysis to provide a more focused examination of CC. The findings will help provide insights for policy changes to improve access to CC screening and promote culturally competent care.

## 2. Materials and Methods

### 2.1. Data Sources

The primary data for this study were obtained from the 2017–2021 MEPS administered by the Agency for Healthcare Research and Quality. Data before this time period were not used due to the survey’s redesign in 2017 [13]. The MEPS is a national survey of noninstitutionalized individuals used to study healthcare use, expenditures, and sources of payment while collecting information on demographics and socioeconomic status. The MEPS uses stratification, clustering, and multiple stages of selection to form its nationally representative survey pool, and its method to calculate standard errors is available online [14]. To obtain accurate estimates from MEPS data, each yearly consolidated file includes the individual’s weight, stratum, and primary sampling unit (PSU) variables.

To examine factors related to age-adjusted CC rates, data on county-level social determinants of health were drawn from the 2017–2021 County Health Rankings (CHR). The CHR is an annual database that measures social, behavioral, and clinical factors for each county in all 50 states of the United States and the District of Columbia [15]. To merge the data from both the MEPS and CHR, we submitted a request for confidential data files and used “county” as a linkage term to merge the two datasets. After the merger, all geographic information was removed again to protect individual privacy. Our study focused exclusively on CC among the AA population. Rectal cancer was not analyzed in this study, and since it is classified as a rare disease, it was excluded from the dataset by the MEPS to safeguard patient privacy.

### 2.2. Study Sample

This study focused on adults aged 18 and older who self-reported their demographics and health conditions in the MEPS. The unweighted sample size of adults was 23,733 in 2017, 23,027 in 2018, 21,965 in 2019, 21,879 in 2020, and 22,785 in 2021, and of them, there were 1501, 1217, 1149, 1176, and 1163 AA adults from 2017 to 2021. Thirteen cancer questions were asked, and only those who responded “Yes” to colon cancer were considered our target population. The unweighted sample size of adults with CC was 133 in 2017, 114 in 2018, 129 in 2019, 147 in 2020, and 154 in 2021.

### 2.3. Measurements

Our outcomes included the crude CC rate and age-adjusted rate. Two Supplementary Tables (Appendix A) provide the denominators and numerators used to calculate the age-specific rates. We first divided the full sample by every 10 years of age to obtain the ratio of AAs in each age group in each year from 2017 to 2021 (Formula (1)). The adjustment was accomplished by multiplying the age-specific rates of AAs with CC by the age-specific weights among all AAs (Formula (2)). Finally, we summed all the age-specific rates across the six age groups to yield the final age-adjusted rate for each of the five years (Formula (3)).

Age-Specific Rate = (Number of AAs for each age group ÷ Number of AAs) × 100%(1)

Crude CC Rate = (Number of AAs with CC for each age group ÷ Number of AAs with CC) × 100%(2)

Age-Adjusted CC Rate = Σ (Crude Rate × Age-Specific Rate)(3)

Our first independent variable, “Year”, was used to examine the trend of crude CC and adjusted rates among AAs over five years. Our second independent variable, “State”, was used to identify which state has the highest number of AAs with CC. Our third risk factor included two individual-level characteristics: whether the survey respondent spoke English or another language and whether the respondent had insurance at the time of the survey. Our last set of risk factors included 12 county-level characteristics from the 2017–2021 CHR data: percentages of older adults, children or adolescents; physical and mental distress; food and housing insecurity; percentage of children in poverty; uninsured and unemployed rates; percentage of Native Americans; and daily PM 2.5 level (a measure of air pollution consisting of particles less than 2.5 μm in diameter). The source and definition of each measurement can be found on the CHR website [15]. Finally, the study tested several other characteristics such as marital status, rural/urban classification, residential segregation index, and health conditions (e.g., high blood pressure) but found no significant differences. Therefore, this study reported only the 14 factors that yielded significant results.

### 2.4. Statistical Analysis

We first examined yearly trends in the percentage of people with CC in five racial/ethnic groups: White, Black, Hispanic, Asian, and Other. Weighted frequency, row percentages, and column percentages were used to examine intra-racial and inter-racial changes over five years. Next, we adjusted the crude prevalence rate of CC by each year’s age distribution. The age-specific rate was obtained for every 10 years of age, and the age-adjusted rate was obtained by summing all 6 age groups’ rates. Third, we presented the age-adjusted rate and standard deviation of AAs with CC in each of the 50 states. We conducted linear regression using the state of Alaska as the reference group to determine if each state’s rate was significantly higher than that of Alaska. Finally, we compared CC rates using two personal-level characteristics and twelve county-level characteristics. T tests were used for categorical variable risk factors (e.g., insured vs. uninsured), and regression models were used for continuous variable risk factors (e.g., unemployment rate). Variance and standard errors were estimated using the Taylor-series linearization method according to MEPS’s user guideline [16]. All analyses were carried out by STATA v18 (StataCorp, Inc., College Station, TX, USA), and any *p*-value less than 0.05 was considered statistically significant.

## 3. Results

Table 1 shows the numbers and percentages of adults with CC by race/ethnicity and year. For each racial group, we summed the yearly number and calculated the yearly percentage by dividing each year’s number by the total number of adults with CC in that particular group (inter-racial percentages). Similarly, for each year, we summed the total number of adults with CC across five racial groups and calculated the intra-racial percentage by dividing each race’s number by the total number of adults with CC in one particular year. There were 377,995 AA adults with CC over the five years, and the column percentages showed that the CC rate within the AA group increased from 6.1% to 31.8% from 2017 to 2021. Next, the weight sample size remained plateaued, from 1,451,992 in 2017 to 1,388,760 in 2021. However, the row percentages showed that the percentage of AAs in the CC populations increased from 1.6% in 2017 to 8.6% in 2021. We also found increases in the percentages of Native Americans and multiracial groups with CC, but the differences across the five racial groups were not statistically significant (*p* = 0.1689).

Table 2 provides the percentage of AAs in the population as well as the proportion of AAs with CC within each age group per year. Because age is a significant risk factor for having any illness [17,18], we adjusted each prevalence by the yearly age distribution and obtained an age-adjusted rate of CC per year, which increased from 155 per 100,000 in 2017 to 753 per 100,000 in 2021, which is approximately 4.86 times higher.

Table 3 compares the age-adjusted rate among the 50 states and the District of Columbia by using the state of Alaska (AL) as the reference group. We found that Arkansas (AR = 0.00716), New Hampshire (NH = 0.00691), and Rhode Island (RI = 0.00691) had significantly higher rates of CC prevalence, while South Carolina (SC = 0.00248), Vermont (VT = 0.00256), and Wyoming (WY = 0.00228) had significantly lower rates than Alaska (*p* < 0.05). Among the 51 regions, AAs in Arkansas had the highest CC prevalence rate, at 716 per 100,000, while those in Wyoming had the lowest rate, at 228 per 100,000, representing approximately a 3.14-fold difference between the two states.

**Figure 1:**
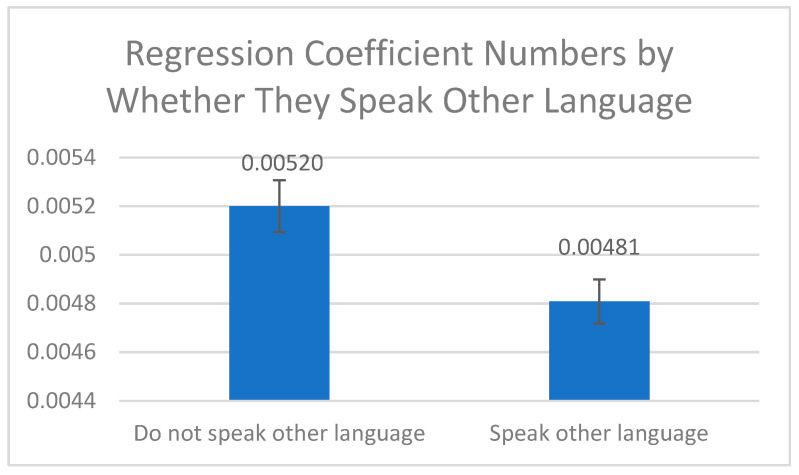
Association between language spoken (other than English) and age-adjusted colon cancer rate.

The three figures below show the differences in the age-adjusted rates and standard deviations of the 14 characteristics found to be statistically significant. Figure 1 illustrates that the age-adjusted rates were positively associated with AAs speaking English (mean: 520 > 480 per 100,000, *p* = 0.001). No significant difference was found in CC rates between American-born and foreign-born AAs. However, Figure 1 implies that people who speak other languages may be exposed to different diets and non-Western medicine practices, which may act as a protective factor.

**Figure 2:**
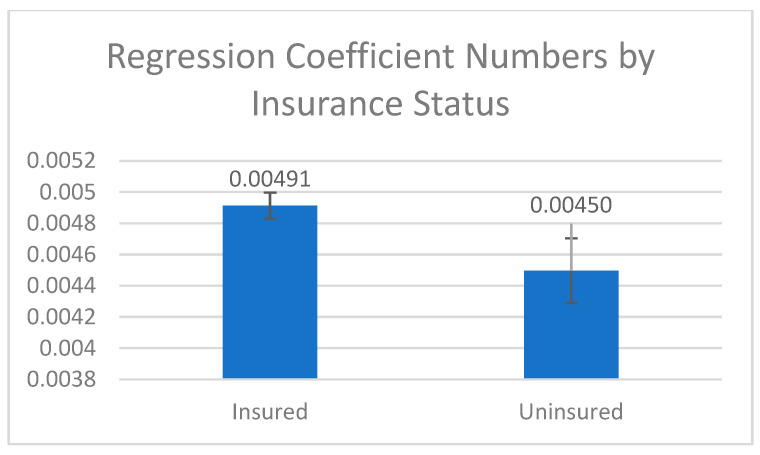
Association between insurance status and age-adjusted colon cancer rate.

Figure 2 shows that the age-adjusted CC rate was positively associated with health insurance status (mean: 491 > 450 per 100,000, *p* = 0.039), implying that AAs with health insurance are more likely to have access to CC screening and detection.

**Figure 3:**
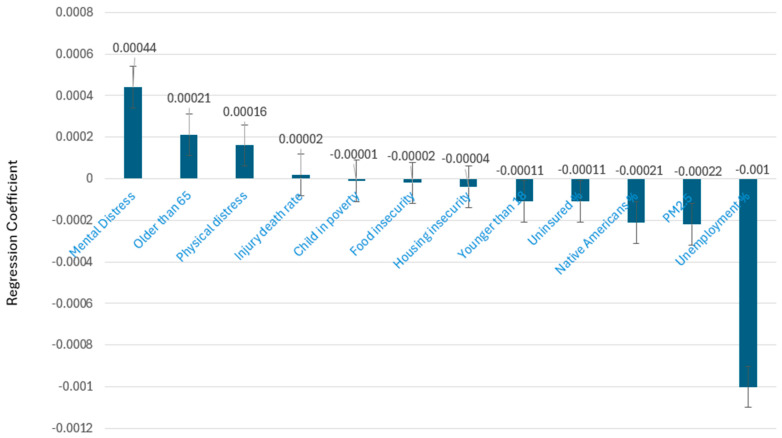
Association between county-level factors and age-adjusted colon cancer rate.

Figure 3 illustrates the 12 county-level characteristics with the age-adjusted CC rate and 95% Confidence Interval. The age-adjusted rate of CC was positively associated with four factors: a higher percentage of people with mental distress, a higher percentage of people aged 65 and older, a higher percentage of people with physical distress, and a higher injury-related death rate (*p* < 0.05). Meanwhile, the age-adjusted rate of CC was negatively associated with eight factors: a higher percentage of children in poverty, a higher percentage of food insecurity, a higher percentage of housing insecurity, a higher percentage of people aged 18 and younger, a higher percentage of uninsured individuals, a higher percentage Native Americans or Native Alaskans, higher average daily PM 2.5, and a higher percentage of unemployment (*p* < 0.05). This finding suggests that CC prevalence is related to access to preventive care and CC screening. Areas with a lower socioeconomic status may have less access to screening and therefore have a lower CC prevalence than more wealthy areas where screening is more accessible.

## 4. Discussion

### 4.1. Increasing Trend of Age-Adjusted Colon Cancer (CC) Rates

Our study provides a comprehensive analysis of CC prevalence among AAs, revealing a concerning trend of rising rates. The annual CC prevalence increased significantly, from 6.1% in 2017 to 31.8% in 2021, a rise potentially driven by either a rise in CC risk factors or more widespread adoption of CC screening practices. The proportion of CC prevalence among AAs compared to the general population rose from 1.6% to 8.6% during this period, and the age-adjusted CC prevalence rate increased from 155 per 100,00 to 753 per 100,000, as shown in Table 2. While prevalence statistics are not readily available, a previous study on colorectal cancer from 2015 to 2019 in Asian Americans and Pacific Islanders found an incidence rate of 33.9 per 100,000 for men and 24.3 per 100,00 for women [19]. Our age-adjusted rate accounts for the higher susceptibility of older individuals to cancer, making the stable increase concerning, especially considering that AA patients are more likely than White patients to present with advanced stages of CC and experience longer delays before surgery [20]. Understanding these trends will be crucial for developing targeted interventions to address the rising CC rates in the AA population and improve outcomes.

Our study is the first to use MEPS and CHR data to analyze CC prevalence at the state and county levels among AAs, one of the fastest-growing minority groups. Cultural factors may influence screening behaviors in AAs. One study found that Chinese and Korean immigrants were less likely to undergo colonoscopies due to time constraints or lack of symptoms, and preventive healthcare is not prioritized by many who believe they can manage their health through diet and exercise [21]. The perception of colon cancer as a predominantly “Western disease” further reduces the urgency of screening among AA immigrants [21]. Collectivist values may lead some to avoid screening to prevent burdening their families with a potential diagnosis [21]. More acculturated AAs have higher screening rates, likely due to their familiarity with the healthcare system [11]. Social factors such as being married and receiving positive feedback about screening from friends and family also contribute to greater openness to screening [11]. Future studies should continuously monitor the screening rate among AAs.

### 4.2. State-Level Variations in CC Rates

State-level CC prevalence data highlight potential socioeconomic and geographical disparities in healthcare access. Arkansas (716 per 100,000), Rhode Island (691 per 100,000), and New Hampshire (691 per 100,000) had the highest CC prevalence rates among AAs. Arkansas’ high rate is likely influenced by lifestyle and dietary factors typical of the South [22]. However, the high prevalence in Rhode Island and New Hampshire, both located in New England, suggests that other factors may be at play. One theory proposes that reduced sunlight exposure in more northern regions contributes to vitamin D deficiency, which is linked to increased colorectal cancer risk [23]. Additionally, these three states had AA median household incomes (MHIs) well above the state averages [24], a factor associated with better healthcare access and enhanced screening rates, and thus more detected cases of CC [25].

In contrast, Wyoming (228 per 100,000), South Carolina (248 per 100,000), and Vermont (256 per 100,000) had the lowest CC prevalence rates among AAs. The reasons for these lower rates are not entirely clear, although geographic and demographic factors may provide some explanation. These states, especially Wyoming, have barriers to healthcare access due to their predominantly rural nature. Wyoming’s sparsely populated regions face challenges in primary care availability and affordability [26], thus contributing to decreased screening rates [27]. Additionally, all three states have relatively small AA populations that are generally dispersed across small towns and cities, limiting the formation of ethnic enclaves and access to culturally sensitive healthcare services that promote screenings [28]. More research is needed to understand the specific factors driving these regional disparities.

### 4.3. Personal Characteristics Associated with CC Rates

The higher CC prevalence found in our study among English-only speaking AAs and those with health insurance may be largely attributed to increased access to screening and, thus, detection of cancer [10,29,30,31]. English proficiency is a significant protective factor for CC screening among AAs, as those with high English proficiency often experience enhanced communication with healthcare providers, increased knowledge of CC, and greater compliance with screening guidelines [10,29,32,33,34,35]. In contrast, lower acculturation levels and limited English proficiency are linked to a decline in the perceived quality of healthcare services and decreased health literacy, resulting in lower CC screening rates [36]. Efforts to improve colorectal cancer screening in AAs by addressing the need for culturally sensitive care have been met with mixed results. A pilot program for culturally sensitive education on colorectal cancer screening from community health workers did not significantly change screening intentions among Chinese, Korean, and Vietnamese adults [37]. However, bilingual health educators increased colorectal cancer screening rates in Hmong Americans, suggesting that culturally sensitive care can be effective in populations with limited English proficiency [38]. Therefore, English proficiency among AAs is a key factor that facilitates better communication with healthcare providers and adherence to screening guidelines. For people without bilingual capacity, translation services would help promote higher screening rates and potentially greater detection of CC.

Health insurance is another significant protective factor in CC prevention, as it increases access to preventive care and screenings. Early detection is critical, with the U.S. Preventative Services Task Force advising colorectal cancer screening for all adults aged 45 to 75 utilizing stool-based tests (e.g., guaiac fecal occult blood test, fecal immunochemical test, and stool DNA test) and direct visualization tests (e.g., colonoscopy, CT colonography, and flexible sigmoidoscopy) [39,40]. Lack of insurance is a known barrier to CC screening, as uninsured individuals are less likely to undergo regular preventive visits and routine screenings [30,31]. However, even people with insurance may not obtain full coverage for screening [41]. For example, colorectal cancer screening rates remain lower among insured AAs in California compared to insured White Americans (54.9% vs. 59.4%), despite expanded coverage under the Affordable Care Act [42]. Overall, health insurance coverage significantly enhances CC screening rates among AAs, although more targeted interventions are necessary to close the remaining gaps.

### 4.4. County-Level Characteristics Associated with CC Rates

Our study also measured 12 county characteristics that support the existing literature linking socioeconomic status to CC screening. Counties with higher levels of personal and health distress, older populations, and higher injury death rates also had higher CC prevalence. These factors suggest that counties facing greater stressors and older populations may be subject to more risk factors, leading to higher CC rates. While older age is a known risk factor for colon cancer, it is also associated with increased screening [30]. Individuals in these counties may also be likely to have regular visits to primary care providers, a factor positively associated with increased CC screening in AAs [30,43].

Counties with higher levels of poverty, food insecurity, housing insecurity, uninsured status, exposure to PM 2.5, and unemployment had lower CC prevalence, likely reflecting reduced access to preventive healthcare services, resulting in fewer reported CC cases. The American Cancer Society reports that only 21% of uninsured adults aged 45 and older in the general population received colorectal cancer screening, with lower-income adults showing similar trends [6]. Higher MHIs contribute to improved healthcare, earlier detection, timely treatment, better quality of care, and better long-term survival rates [25]. Additionally, greater MHIs influence non-healthcare factors such as better access to education, healthier lifestyles, and reduced environmental exposures [24]. This association highlights the socioeconomic barriers that contribute to CC prevalence.

### 4.5. Limitations

Our study has some limitations. First, a study using 2009–2014 national data showed that Filipinos had a higher screening rate (55.0%) than Chinese (50.0%) or Asian Indian populations (48.6%) [30]. However, our data do not disaggregate Asian subgroups, of which there are at least 21 with distinct cultural and dietary practices. Second, the vast cultural and socioeconomic differences between states were not fully explored in this analysis. Our study could provide an analysis of only county- and personal-level risk factors. Thirdly, the data from the MEPS and CHR are self-reported and may be subject to recall bias. While validation with claims data could address this, this was not feasible in our study due to the confidentiality of the dataset. Claims data are restricted to individuals who generate claims, which could potentially lead to the exclusion of cases where healthcare services were not sought for colon cancer during the five-year period. Fourthly, institutionalized patients with CC were not included, likely leading to an underestimation of the prevalence of CC in the population studied. Fifthly, our study does not include survival data, limiting our ability to assess how advancements in early detection and timely treatment may have influenced prevalence rates. Overall survival has increased over time, driven by early-stage diagnosis and timely treatment initiation within 60 days of diagnosis, likely contributing to the increased prevalence [44]. Lastly, CC is often grouped with rectal cancer under the umbrella term of colorectal cancer. Since rectal cancer is considered a rare disease, it was removed by the MEPS from the data to protect patient privacy. Thus, the findings of this study should be interpreted within the context of CC only. Many of the studies referenced in this paper focus on colorectal cancer without disaggregating colon and rectal cancer. This limitation underscores the need for targeted research on CC specifically within AA populations.

## 5. Conclusions

Our study demonstrated the rising incidence of colon cancer in AAs since 2017. As the third most commonly diagnosed cancer in the United States, it is critical to understand the contributing factors. Our study found that colon cancer is more likely to be diagnosed in AA populations who have increased access to care and familiarity with medical screenings. Future studies should determine whether the early diagnosis of CC in AA communities leads to better outcomes and earlier intervention/access to treatment. To address the rising rate of CC diagnosis in the AA community, we also need to address the barriers to accessing routine preventive screening measures.

## Figures and Tables

**Table 1:** Weighted racial distribution of adults with colon cancer by year.

Year (weighted n, %)	White (N = 4,982,631)	Black (N = 578,209)	Hispanic (N = 536,967)	Asian (N = 377,995)	Other (N = 300,645)	Total (N = 6,776,447)
2017	1,087,380	159,094	162,341	23,209	19,968	1,451,992
(col%)	(21.8%)	(27.5%)	(30.2%)	(6.1%)	(6.6%)	(21.4%)
(row%)	(74.9%)	(11.0%)	(11.2%)	(1.6%)	(1.4%)	(100.0%)
2018	818,927	71,124	130,149	51,367	61,828	1,133,395
(col%)	(16.4%)	(12.3%)	(24.2%)	(13.6%)	(20.6%)	(16.7%)
(row%)	(72.3%)	(6.3%)	(11.5%)	(4.5%)	(5.5%)	(100.0%)
2019	1,017,906	100,888	73,796	77,814	65,211	1,335,614
(col%)	(20.4%)	(17.4%)	(13.7%)	(20.6%)	(21.7%)	(19.7%)
(row%)	(76.2%)	(7.6%)	(5.5%)	(5.8%)	(4.9%)	(100.0%)
2020	1,100,770	116,334	78,821	105,496	65,264	1,466,686
(col%)	(22.1%)	(20.1%)	(14.7%)	(27.9%)	(21.7%)	(21.6%)
(row%)	(75.1%)	(7.9%)	(5.4%)	(7.2%)	(4.4%)	(100.0%)
2021	957,648	130,769	91,860	120,109	88,374	1,388,760
(col%)	(19.2%)	(22.6%)	(17.1%)	(31.8%)	(29.4%)	(20.5%)
(row%)	(69.0%)	(9.4%)	(6.6%)	(8.6%)	(6.4%)	(100.0%)

Note: *p* = 0.1689. Note: n = frequency; col = column.

**Table 2:** Age-adjusted colon cancer prevalence rate among Asian Americans by year.

Age	<=30	31–40	41–50	51–60	61–70	71+	Age-Adjusted Rate (All Rate × cc Rate)
2017 (all)	0.2465	0.2010	0.2131	0.1372	0.1127	0.0893	0.0893 × 0.0174 = 0.00155
2017 (cc)	0	0	0	0	0	0.0174	
2018 (all)	0.2367	0.2102	0.2129	0.1260	0.1203	0.0939	(0.1203 × 0.0151) + (0.0939 × 0.0162) = 0.00334
2018 (cc)	0	0	0	0	0.0151	0.0162	
2019 (all)	0.2377	0.2147	0.1950	0.1543	0.1024	0.0959	(0.1950 × 0.0058) + (0.1024 × 0.0178) + (0.0959 × 0.0215) = 0.00502
2019 (cc)	0	0	0.0058	0	0.0178	0.0215	
2020 (all)	0.2315	0.1992	0.1960	0.1631	0.1094	0.1008	(0.1094 × 0.0102) + (0.10089 × 0.0565) = 0.00682
2020 (cc)	0	0	0	0	0.0102	0.0565	
2021 (all)	0.2236	0.1950	0.1879	0.1720	0.1088	0.1127	(0.1088 × 0.0136) + (0.1127 × 0.0537) = 0.00753
2021 (cc)	0	0	0	0	0.0136	0.0537	

Note: cc = colon cancer

**Table 3:** Age-adjusted colon cancer rates among Asian Americans in 50 states and the District of Columbia, using Alaska as the reference group.

State	Rate (SD)	State	Rate (SD)	State	Rate (SD)
AL	0.00506 (0.00080)	LA	0.00530 (0.00095)	OH	0.00517 (0.00036)
AK	0.00372 (0.00946)	ME	0.00632 (<0.0001)	OK	0.00582 (0.00004)
AZ	0.00417 (0.00062)	MD	0.00510 (0.00042)	OR	0.00412 (0.00087)
AR *	0.00716 (0.00001)	MA	0.00499 (0.00040)	PA	0.00438 (0.00026)
CA	0.00495 (0.00020)	MI	0.00441 (0.00055)	RI *	0.00691 (0.00049)
CO	0.00519 (0.00026)	MN	0.00449 (0.00023)	SC *	0.00248 (0.00041)
CT	0.00627 (0.00021)	MS	0.00502 (<0.0001)	SD	0.00619 (<0.01001)
DE	0.00593 (<0.0001)	MO	0.00524 (0.00069)	TN	0.00516 (0.00103)
FL	0.00534 (0.00025)	MT	0.00583 (<0.0001)	TX	0.00509 (0.00020)
GA	0.00468 (0.00024)	NE	0.00582 (0.00053)	UT	0.00463 (0.00049)
HI	0.00485 (0.00040)	NV	0.00519 (0.00026)	VT *	0.00256 (0.00074)
ID	0.00472 (0.00130)	NH *	0.00691 (0.00025)	VA	0.00473 (0.00029)
IL	0.00482 (0.00024)	NJ	0.00491 (0.00040)	WA	0.00446 (0.00031)
IN	0.00523 (0.00031)	NM	0.00512 (0.00014)	WV	-
IA	0.00540 (0.00071)	NY	0.00487 (0.00019)	WI	0.00564 (0.00034)
KS	0.00492 (0.00127)	NC	0.00429 (0.00042)	WY *	0.00228 (<0.0001)
KY	0.00386 (0.00096)	ND*	0.00258 (<0.0001)	DC	0.00553 (0.00062)

Note: SD = standard error; *: *p* < 0.05, meaning they have a significantly higher or lower rate than Alaska (reference group).

## Data Availability

The Medical Expenditure Panel Survey contains de-identified data available on the website of the Agency for Healthcare Research and Quality: https://meps.ahrq.gov/mepsweb/data_stats/download_data_files.jsp (accessed on 30 March 2024). However, confidential data, including rural–urban categories and detailed ICD-10 codes, are only available through applications submitted to the Center for Financing, Access, and Cost Trends Data Center (CFACT Data Center). The application for the confidential data can be found online: https://meps.ahrq.gov/mepsweb/data_stats/onsite_datacenter.jsp (accessed on 30 March 2024).

## Appendix A

**Table A1:** Unweighted Sample Size by Year and Race/Ethnicity.

Year	White	Black	Hispanic	Asian	Others	Total
2017 (all)	11,461	3987	6052	1501	732	23,733
2017 (cc)	85 (0.74%)	21 (0.53%)	19 (0.31%)	<11 (<1.00%)	<11 (<1.00%)	133 (0.56%)
2018 (all)	12,796	3403	4866	1217	745	23,027
2018 (cc)	78 (0.61%)	12 (0.35%)	12 (0.25%)	<11 (<1.00%)	<11 (<2.00%)	144 (0.50%)
2019 (all)	12,534	3130	4462	1149	690	21,965
2019 (cc)	92 (0.73%)	17 (0.54%)	<11 (<1.00%)	<11 (<1.00%)	<11 (<1.00%)	129 (0.59%)
2020 (all)	12,217	3065	4791	1176	630	21,879
2020 (cc)	187 (0.88%)	17 (0.55%)	11 (0.23%)	<11 (<1.00%)	<11 (<1.00%)	147 (0.67%)
2021 (all)	12,808	3273	4856	1163	388	22,785
2021 (cc)	106 (0.83%)	21 (0.64%)	11 (0.23%)	<11 (<1.00%)	<11 (<1.00%)	154 (0.68%)

Note: cc = colon cancer.

**Table A2:** Weighted sample size of Asian Americans by year and age category (numbers used to calculate age-adjusted rates given in Table 2).

	Age (in Years)					
Year	<30	31–40	41–50	51–60	61–70	71+
2017 (all)	3,690,366	3,009,523	3,190,829	2,054,585	1,687,733	1,337,459
2017 (cc)	0	0	0	0	0	23,209
2018 (all)	3,647,937	3,239,001	3,281,121	1,942,190	1,853,426	1,447,776
2018 (cc)	0	0	0	0	27,965	23,401
2019 (all)	3,691,646	3,334,173	3,028,825	2,396,794	1,591,024	1,490,186
2019 (cc)	0	0	17,425	0	28,315	32,073
2020 (all)	3,587,151	3,086,144	3,036,222	2,526,148	1,695,181	1,561,851
2020 (cc)	0	0	0	0	17,280	88,217
2021 (all)	3,562,757	3,106,470	2,993,299	2,740,715	1,733,848	1,795,663
2021 (cc)	0	0	0	0	23,660	96,449

Note: cc = colon cancer.
