# Correction: Do et al. Colon Cancer Rates Among Asian Americans: A 2017–2021 Epidemiological Analysis. *Cancers* 2024, *16*, 4254

**DOI:** 10.3390/cancers17223700

**Published:** 2025-11-19

**Authors:** Candice Do, Wei-Chen Lee, Christopher Huy D. Doan, Cathy Z. Xie, Kendall M. Campbell

**Affiliations:** 1 John Sealy School of Medicine, University of Texas Medical Branch, Galveston, TX 77555, USA; cado@utmb.edu (C.D.); cddoan@utmb.edu (C.H.D.D.); 2 Department of Family Medicine, University of Texas Medical Branch, Galveston, TX 77555, USA; czxie@utmb.edu (C.Z.X.); kemcampb@utmb.edu (K.M.C.)

The authors would like to make a number of corrections to their published paper [1].

In the published version of this article, the Simple Summary included the following statement: “Communities with greater socioeconomic resources had lower rates of colon cancer, likely due to better access to early screenings”. However, the statement should be corrected to read as follows: “Communities with greater socioeconomic resources had higher rates of colon cancer, likely due to better access to early screenings”. The corrected Simple Summary is included below.

- **Simple Summary:** Colon cancer is an increasing health concern, especially for Asian Americans. Our study analyzed recent data to understand colon cancer prevalence and risk factors in Asian Americans across different states and counties. We found that colon cancer prevalence among Asian Americans increased significantly between 2017 and 2021, and the highest rates were in Arkansas, Rhode Island, and New Hampshire. Speaking only English and having health insurance were associated with higher prevalence of colon cancer, possibly due to greater access to screening. Communities with greater socioeconomic resources had higher rates of colon cancer, likely due to better access to early screenings. These findings highlight the importance of preventive efforts to address the increased colon cancer rates in the Asian American population. Future studies should examine geographic and community-level factors contributing to these disparities.

The Abstract in the published version of the article included a sentence stating that “County-level analysis identified lower CC prevalence in regions with a greater socioeconomic advantage (*p* < 0.05)”. However, this statement should be corrected to the following: “County-level analysis identified higher CC prevalence in regions with greater socioeconomic advantage (*p* < 0.05)”. The corrected Abstract is included below.

- **Abstract: Background:** Colon cancer (CC) is a significant public health concern. With Asian Americans (AA) representing a rapidly growing demographic in the United States, our study examined CC prevalence among AA. **Methods:** The study merged 2017–2021 Medical Expenditure Panel Survey and County Health Ranking. Our analysis calculated age-adjusted CC rates and examined prevalence across states. Regression analyses were conducted to study county-level risk factors of CC. **Results:** The CC age-adjusted rate among AA increased five-fold, from 155 per 100,000 in 2017 to 753 per 100,000 in 2021. State-level disparities revealed the highest CC prevalence in Arkansas, Rhode Island, and New Hampshire. Not speaking other languages and having insurance were significantly associated with higher CC rates, suggesting barriers to preventions and higher use of screening (*p* < 0.05). County-level analysis identified higher CC prevalence in regions with greater socioeconomic advantage (*p* < 0.05). Socioeconomic advantage seemed to facilitate higher screening rates, translated into higher CC rates. **Conclusions:** Our findings underscore the need for early preventions to address rising CC rates among AA. Future research should also explore geographic to better understand the disparities in CC risk.

In the online version of the article, Table A1 includes data values of “11”, which should be replaced with “<11”. The corresponding percentage values have also been adjusted to “<1.00%” and “<2.00%”. The authors would like to replace Table A1 with a new corrected version, which is included below.

**Table A1:** Unweighted Sample Size by Year and Race/Ethnicity.

Year	White	Black	Hispanic	Asian	Others	Total
2017 (all)	11,461	3987	6052	1501	732	23,733
2017 (cc)	85 (0.74%)	21 (0.53%)	19 (0.31%)	<11 (<1.00%)	<11 (<1.00%)	133 (0.56%)
2018 (all)	12,796	3403	4866	1217	745	23,027
2018 (cc)	78 (0.61%)	12 (0.35%)	12 (0.25%)	<11 (<1.00%)	<11 (<2.00%)	144 (0.50%)
2019 (all)	12,534	3130	4462	1149	690	21,965
2019 (cc)	92 (0.73%)	17 (0.54%)	<11 (<1.00%)	<11 (<1.00%)	<11 (<1.00%)	129 (0.59%)
2020 (all)	12,217	3065	4791	1176	630	21,879
2020 (cc)	187 (0.88%)	17 (0.55%)	11 (0.23%)	<11 (<1.00%)	<11 (<1.00%)	147 (0.67%)
2021 (all)	12,808	3273	4856	1163	388	22,785
2021 (cc)	106 (0.83%)	21 (0.64%)	11 (0.23%)	<11 (<1.00%)	<11 (<1.00%)	154 (0.68%)

Note: cc = colon cancer.

In the published version of the article, the authors would like to add a statement acknowledging AHRQ and the CFACT Data Center. The revised acknowledgements statement is included below.

- **Acknowledgments:** The research in this paper was conducted at the CFACT Data Center, and the support of AHRQ is acknowledged. The results and conclusions in this paper are those of the authors and do not indicate concurrence by AHRQ or the Department of Health and Human Services.

With this correction, the name of the author “Cathy Z. Xie” has also been updated to include the middle name initial. The authors state that the scientific conclusions are unaffected. This correction was approved by the Academic Editor. The original publication has also been updated.
